# Cancer prevention through weight control—where are we in 2020?

**DOI:** 10.1038/s41416-020-01154-3

**Published:** 2020-11-25

**Authors:** Annie S. Anderson, Andrew G. Renehan, John M. Saxton, Joshua Bell, Janet Cade, Amanda J. Cross, Angela King, Elio Riboli, Falko Sniehotta, Shaun Treweek, Richard M. Martin, On behalf of the UK NIHR Cancer and Nutrition Collaboration (Population Health Stream), Annie Anderson, Rebecca Beeken, Janet Cade, Amanda Cross, Angela King, Richard Martin, Giota Mitrou, Elio Riboli, John Saxton, Andrew Renehan

**Affiliations:** 1 Centre for Research into Cancer Prevention and Screening, Division of Population Health & Genomics. Level 7, Mailbox 7, University of Dundee, Ninewells Hospital and Medical School, Dundee, DD1 9SY UK; 2 The Christie NHS Foundation Trust, Manchester Cancer Research Centre, NIHR Manchester Biomedical Research Centre, Division of Cancer Sciences, School of Medical Sciences, Faculty of Biology, Medicine and Health University of Manchester, Wilmslow Rd, Manchester, M20 4BX UK; 3 Department of Sport, Exercise & Rehabilitation, Faculty of Health and Life Sciences, Northumbria University, Room 259, Northumberland Building, Newcastle Upon Tyne, NE1 8ST UK; 4 MRC Integrative Epidemiology Unit, University of Bristol, Oakfield House, Bristol, BS8 2BN UK; 5 Nutritional Epidemiology Group, School of Food Science and Nutrition, G11, Stead House, University of Leeds, Leeds, LS2 9JT UK; 6 Department of Epidemiology and Biostatistics, School of Public Health, Imperial College London, Norfolk Place, London, W2 1PG UK; 7 NIHR Cancer and Nutrition Collaboration, Level E and Pathology Block (mailpoint 123), Southampton General Hospital, Tremona Road, Southampton, SO16 6YD UK; 8 Policy Research Unit Behavioural Science, Faculty of Medical Sciences, Newcastle University, Baddiley-Clark Building, Richardson Road, Newcastle upon Tyne, NE2 4AX UK; 9 Health Services Research Unit, University of Aberdeen, Room 306, 3rd Floor, Health Sciences Building, Foresterhill, Aberdeen AB25 2ZD UK; 10 Professor of Public Health Nutrition, Dundee Centre for Research into Cancer Prevention and Screening, London, UK; 11 Yorkshire Cancer Research University Academic Fellow, University of Leeds, London, UK; 12 Professor of Nutritional Epidemiology and Public Health, University of Leeds, London, UK; 13 Professor in Cancer Epidemiology, Imperial College London, London, UK; 14 Public Representative, London, UK; 15 Professor of Clinical Epidemiology, University of Bristol, Bristol, BRC UK; 16 Director of Research Funding & Science External Relations, WCRF, London, UK; 17 Chair in Cancer Epidemiology and Prevention, Imperial College London, London, UK; 18 Professor in Clinical Exercise Physiology, Northumbria University, London, UK; 19 Professor of Cancer Studies and Surgery, University of Manchester; Honorary Consultant Colorectal Surgeon, London, UK

**Keywords:** Lifestyle modification, Cancer prevention, Public health

## Abstract

Growing data from epidemiological studies highlight the association between excess body fat and cancer incidence, but good indicative evidence demonstrates that intentional weight loss, as well as increasing physical activity, offers much promise as a cost-effective approach for reducing the cancer burden. However, clear gaps remain in our understanding of how changes in body fat or levels of physical activity are mechanistically linked to cancer, and the magnitude of their impact on cancer risk. It is important to investigate the causal link between programmes that successfully achieve short-term modest weight loss followed by weight-loss maintenance and cancer incidence. The longer-term impact of weight loss and duration of overweight and obesity on risk reduction also need to be fully considered in trial design. These gaps in knowledge need to be urgently addressed to expedite the development and implementation of future cancer-control strategies. Comprehensive approaches to trial design, Mendelian randomisation studies and data-linkage opportunities offer real possibilities to tackle current research gaps. In this paper, we set out the case for why non-pharmacological weight-management trials are urgently needed to support cancer-risk reduction and help control the growing global burden of cancer.

## Background

Cancer causes one in six deaths globally and is now overtaking cardiovascular disease as the leading cause of death across much of the world.^1,2^ Currently, tobacco use is the most important single modifiable risk factor for cancer, but obesity (and its determinants—high intakes of energy-dense, ultra-processed foods and drinks, and low levels of physical activity) is becoming increasingly visible as the second most common cause of cancer. According to the World Health Organisation (WHO), 1.9 billion adults and over 340 million children and adolescents were living with overweight or obesity in 2016 (i.e. a body mass index (BMI) > 25 kg/m^2^) and these numbers are projected to rise.^3^ This situation is compounded by global physical activity data suggesting that more than a quarter of the world’s population is insufficiently active.^4^ Furthermore, overweight and obesity are occurring at earlier ages,^3^ thereby increasing lifetime exposure to associated risks. Current estimates suggest that overweight and obesity could overtake smoking as the single biggest cause of cancer in UK women in around 25 years^5^ and this premise is also echoed in international reports.^6^ Of all new global cancer cases in 2012, 481,000 (or 3.6%) were considered to be attributable to excess body mass index (BMI).^7^

The substantial reduction in lung cancer incidence in countries where public health initiatives have brought about a significant decrease in smoking indicates the potential of primary cancer prevention by societal interventions. The implementation of equitable, population-wide programmes for obesity prevention and management is eagerly awaited, but sufficient evidence already currently exists to justify a research focus on intentional weight loss and cancer-risk-reduction trials. The ultimate objective of trials with positive results must be to create further leverage for the development and implementation of policies aimed at improving the health of the general public—not just the individuals who have the resources and motivation to participate in individually focussed weight-loss programmes.

Pharmaceutical options are available to reduce the risk of obesity-related diabetes and heart disease, but the portfolio of agents that reduce the risk of developing cancer is very limited. Considerable amounts of data, including evidence from randomised controlled trials, support the role of aspirin and tamoxifen in reducing colorectal cancer and breast cancer risk, respectively, and, although further studies also support a role for other drugs, such as metformin^8,9^ and statins,^10^ in cancer prevention, the evidence is much weaker. The effectiveness of these pharmaceuticals is relatively modest compared with drugs available for treating cardiovascular risk factors (hypercholesterolaemia, hypertension and insulin resistance/hyperglycaemia). In addition, the mechanisms of action of these potential cancer-preventive agents are not well-established, and their pleiotropic and undesirable side effects must be considered^11^ alongside evidence of inverse associations with mortality.^12^

Based on the disappointing results of a number of cancer chemoprevention trials conducted over the past three decades,^13^ it is difficult to predict how long it will take to identify effective drugs with low risk of side effects, and we cannot afford to wait for pharmacological approaches alone to prevent cancer risk. The benefit to potentially affected individuals and their families and the direct and indirect economic implications of cancer-risk reduction are far-reaching. Addressing cancer prevention beyond pharmacological solutions has therefore become a global imperative, and strategies that offer disease reduction should no longer be ignored. We now have the evidence to demonstrate that intentional weight loss and weight management as well as increasing physical activity offer much promise as cost-effective approaches for reducing the risk of developing cancer.

## Obesity and cancer

The association between obesity and cancer has been reported and discussed in the literature since the early part of the 20th century.^14^ As population rates of overweight and obesity continue to rise, so will the incidence of common cancers linked to excess body fat (EBF). As a consequence, escalating costs attributable to future cancer treatments and the long-term clinical management of associated comorbidities will place an unrelenting economic burden on healthcare systems. Action needs to be taken now, otherwise our failure to seriously address this topic will leave a sad legacy for the next generation.

### Evidence of an association between excess body fatness and cancer

There is a strong need to address the role of EBF in early life, as it has been demonstrated to influence the risk of many diseases, including cancer, in adulthood. Hidayat et al.^15^ reported associations between body fatness at a young age and the development in later life of eight types of cancer. Jensen et al.^16^ subsequently reported from the Copenhagen School Health Records Registry that children who were heavier or gaining more weight than average at 7–13 years of age (*n* = 257,623) had a significantly greater risk of adult colon cancer.

In adulthood, it seems that although the link between obesity and cancer is becoming more apparent, the significance of weight gain across adult life remains largely ignored. Not only is weight gain the pathway to overweight and obesity, but it is also an independent risk factor for postmenopausal breast cancer risk (~6% per 5-kg increase in adult weight^17^), which is probably most relevant in women with a body mass index (BMI) < 23.4 kg/m^2^ at age 20 (who are more likely to gain weight in adulthood than women with a BMI > 23.4 kg/m^2^).^18^

The latest (2018) World Cancer Research Fund (WCRF)/American Institute for Cancer Research (AICR) expert report^17^ concluded that being overweight or obese throughout adulthood increases the risk of cancers of the mouth, pharynx, larynx, oesophagus (adenocarcinoma), stomach (cardia), pancreas, gall bladder, liver, colorectum, breast (postmenopausal), ovary, endometrium, prostate (advanced) and kidney. In addition, a WHO International Agency for Research on Cancer (IARC) Working Group found evidence relating EBF to meningioma, thyroid cancer and multiple myeloma,^19^ and a hospital-based Danish study of 313,221 patients reported overweight and obesity being related to haematological and neurological cancers.^20^ The reported inverse associations between physical activity and the risk of cancer at 13 sites, including some of the most common cancers (breast, lung, bowel and kidney)^21,22^ reflect the important role of a physically active lifestyle in cancer prevention, either via direct mechanisms, such as improved metabolic control or via its role in the prevention of adult weight gain.^23^ Furthermore, studies show that structured exercise in combination with support for dietary-led weight loss induces more weight loss than exercise or diet alone, and has the greatest impact on blood-borne biomarkers associated with common cancers, including insulin resistance and circulating levels of sex hormones, leptin and inflammatory markers.^24–28^

## Mendelian randomisation studies

In the absence of randomised clinical trials, evidence for causality can be strengthened by Mendelian randomisation (MR) studies.^29^ MR is an instrumental variable method to appraise causality within observational epidemiology, utilising germline genetic variants that are robustly associated with potentially modifiable exposures as proxies (‘instrumental variables’) for the risk factor of interest. As germline genetic variants tend to be randomly distributed with respect to most human traits in the general population, MR studies are less likely to be affected by the sorts of confounding factors that typically bias observational findings. Additionally, as germline genotypes cannot be affected by the presence of disease, the generation of spurious results through reverse causation is avoided. The objective is to identify modifiable intervention targets (behavioural or therapeutic) on the intermediate causal pathway between genetic factors and disease. DNA, although itself unmodifiable, operates through modifiable pathways, e.g., the proprotein convertase subtilisin/kexin type 1 (PCSK1) gene regulates insulin synthesis; fat mass- and obesity-associated (FTO) gene promotes food intake. MR exploits this to identify modifiable exposures that can be used for disease prevention and therapeutic strategies.

Studies using MR support the influence of higher body fatness on greater risk of oesophageal, gastric, pancreatic, renal, colorectal, endometrial and ovarian cancers.^30–33^ Indeed, MR analysis suggests that the obesity-related cancer burden has been substantially underestimated.^34^ The volume and location of fat tissue are strong determinants of insulin resistance and dyslipidaemia, and MR studies support strong effects of higher BMI on higher fasting levels of insulin, glucose, triglycerides, remnant cholesterol and lower high-density lipoprotein (HDL) cholesterol.^35^ The adverse metabolic effects of higher fatness are already evident in late childhood and might worsen with longer time exposure.^36^ Higher body fatness also raises systolic and diastolic blood pressure, and impairs immunity via its association with elevated pro-inflammatory factors such as interleukin-6.^37^ Several of these metabolic traits are associated with an increased risk of obesity-related cancers, with MR evidence being the strongest for higher fasting insulin.^38^

### Excess body fatness and breast cancer risk

It is important to note that, from a life-course perspective, higher body fatness in childhood and adolescence is inversely related to the risk of premenopausal breast cancer as well as postmenopausal breast cancer,^39^ suggesting a long-term protective effect of EBF on breast cancer risk later in life. Analysis from the cohort-pooling project papers^40^ on premenopausal breast cancer confirms that relative overweight at age 18–24 is associated with a modest reduction in the risk of premenopausal breast cancer up to the age of ~50 years, and additional analyses^41^ indicate that weight gain from ages 18–24 to 35–44 or to 45–54 years is also inversely associated with breast cancer overall (e.g., hazard ratio [HR] per 5 kg to ages 45–54: 0.96, 95% confidence interval [CI]: 0.95–0.98) and with oestrogen-receptor(ER)-positive breast cancer (HR per 5 kg to ages 45–54: 0.96, 95% CI: 0.94–0.98).

Evidence related to MR studies also indicates that a genetically predicted larger body size at age 10 might protect against breast cancer in women independent of subsequent body size at a mean age of 56.5 years.^42^ These findings suggest that the effect of early-life body size might persist into later life, regardless of interventions to influence adult body size. There is also evidence^18^ that early-life body size exerts a protective effect even when accounting for age at menarche. A better understanding of the mechanisms linking childhood body size and timing of puberty with later breast cancer risk could help inform potential interventions.

Understanding the crossover effect of obesity with risk reduction before, and risk increase after, menopause is poorly characterised, and further work aimed at understanding the biological mechanisms of how obesity, weight gain and weight change all impact on breast cancer risk is needed.^17^ However, the inverse association of obesity with premenopausal breast cancer does not alter the overall harmful effects of obesity, given that weight and weight gain are positively associated with risks of postmenopausal breast cancer, several other types of cancer and other adverse health outcomes. In addition, women with obesity or who have obesity diagnosed with breast cancer are more likely to have poorer outcomes than leaner women (independent of their menopausal status).^43^

## Weight management—evidence of promise from observational studies

Until 2010, the evidence that intentional weight loss in adulthood modifies cancer risk was sparse, and mostly relied on self-reported body weight with relatively short follow-up periods. However, long-term follow-up data from the Women’s Health Initiative cohort have since reported that, after a mean follow-up of 11.4 years, women with modest weight loss (≥10 pounds from baseline weight during the initial 3-year study) had a lower risk of endometrial cancer compared with those who did not lose weight.^44^ This association was the strongest among women with obesity or who had obesity at baseline. In this cohort, a lower risk of breast cancer among women who lost weight compared with women whose weight remained stable was also reported.^45^ Similarly, the 17-year follow-up of the UK Women’s Cohort Study has shown a lower risk of postmenopausal breast cancer in those individuals who lost weight compared to women with stable weight or those who gained weight.^46^

The largest study to date on weight change and postmenopausal breast cancer is from the Pooling Project of Prospective Studies of Diet and Cancer (DCPP),^47^ which assessed data from 180,885 women aged ≥50 years in whom 6930 invasive breast cancers were identified at the final follow-up. All women were surveyed at three points (baseline, first follow-up (mean of 5.2 years) and final follow-up (10 years)). Sustained weight loss was defined as no less than 2 kg lost between baseline and the first follow-up, which was not regained by the final follow-up. The results demonstrated that, compared with women with stable weight, women with sustained weight loss had a lower risk of breast cancer than women whose weight remained stable; moreover, the larger the weight loss, the lower the risk. It is notable that even modest weight loss (2–4.5 kg) was associated with a significant reduction in risk (HR 0.87, 95% CI 0.77–0.99). Risk reduction was specific to women not using postmenopausal hormone-replacement therapy and the lowest risk was for women who sustained at least 9 kg of weight loss (who were not taking hormone therapy).

## Weight management—indications from intervention studies

Evidence for the impact of weight loss on cancer-risk reduction is also emerging from intervention studies, although no study has yet been designed (in terms of size and follow-up period) specifically to assess the effects of weight loss on cancer incidence or mortality in the general population. Several studies have evaluated the effect of bariatric surgery on cancer risk, comparing people with obesity who underwent surgery with that of individuals in an obesity (non-randomised) control group who did not. According to a systematic review, bariatric surgery was reported to be associated with a reduction in the incidence of overall cancer (pooled odds ratio (POR) = 0.72: 95% CI 0.59–0.87) and in the incidence of obesity-related cancers (POR = 0.55: 95% CI 0.31–0.96).^48^ The cancer-protective effect of bariatric surgery seems to be more pronounced in women than in men, and most marked for a reduction in breast cancer risk. It is notable that the favourable impact of bariatric surgery on cancer risk for adults in mid- and later life occurs within a relatively short follow-up period and is independent of physical activity*.* However, people undergoing bariatric surgery do not necessarily reflect the general overweight and obese population, and the physiological response following acute weight loss might in itself produce effects that might not be matched by weight loss induced through lifestyle interventions.^49^ A systematic review of weight-loss trials^50^ reported a significant reduction in the risk of all-cause mortality, cardiovascular mortality and cancer mortality. Furthermore, in 2020, the Look Ahead Research Group reported^51^ that an intensive lifestyle-intervention trial of 5145 participants, which targeted weight loss, successfully lowered the incidence of obesity‐related cancers by 16% in adults with overweight or obesity and type 2 diabetes after a median follow-up of 11 years, highlighting the potential success of such interventions in cancer-risk reduction.

## Considerations in the design of trials investigating the influence of weight loss on cancer risk

Irrespective of the mode of weight loss, it is important to investigate whether or not programmes that successfully achieve short-term modest weight loss followed by weight-loss maintenance confer benefit on cancer incidence. The potential effect of the latency of risk reduction following weight loss, as well as the duration of overweight and obesity, need to be fully considered in trial design. Furthermore, it is important to identify whether or not the benefits of weight loss are offset by any subsequent regain in weight. There is much to be learnt from highly successful diabetes- prevention programmes based on change in caloric intake and increased physical activity for weight loss,^52,53^ and it is particularly notable that in a 15-year follow-up of the Diabetes Prevention Programme, the incidence of diabetes still remained lower—by 27%—in the lifestyle-intervention group compared with the placebo group.^54^

### The influence of physical activity

Whilst reduced caloric intake plays a greater role than physical activity in weight loss,^55^ the latter might be particularly important in weight-loss maintenance.^56^ However, it is likely that physical activity confers additional benefits on the reduction of cancer risk, for example, through modulation of immune-regulatory pathways,^57^ reduced oxidative stress,^58^ epigenetic changes^59^ and reduced telomere attrition^60^ that may be independent of its effects on body weight.^21^ A 2020 MR study using data from the UK Biobank showed that physical activity is inversely associated with breast and colon cancer risk, independent of its effect on adiposity, and the association between physical activity and cancer incidence at ten sites was shown to be independent of BMI.^61^ Furthermore, strength training, which builds skeletal muscle mass, is inversely associated with the risk of bladder, kidney and colorectal cancer.^62,63^ Improvements in insulin sensitivity and glucose homoeostasis induced by aerobic exercise and/or strength training^64^ could reduce the risk of cancers associated with insulin resistance (and the associated cellular signalling pathways), including cancers of the colon, liver, pancreas and endometrium.^65^

### The influence of dietary factors

Similarly, it is important to consider the independent impact of dietary factors both in terms of macronutrient and micronutrient composition. Strong evidence exists for a protective role of several dietary factors in colorectal cancer (whole grains, foods containing dietary fibre and dairy products) but less so for other cancer sites.^66^ Whilst there has been some promising evidence for the beneficial role of fruit and vegetables in reducing cancer risk, the overall impact on cancer burden is largely limited to cancers of the respiratory and upper digestive tract.^66,67^ Furthermore, enthusiasm for micronutrient supplementation to reduce cancer risk has diminished following a number of randomised control trials that have produced evidence of an associated increased risk of cancer.^68,69^ The lack of the impact of single nutrients/foods on cancer prevention does not mean that the quality of the diet can be ignored. Cancers arising from aberrant metabolic pathways are likely to be influenced by the same nutrients and foods that are associated with the risk of diabetes,^70^ and there is some evidence that healthy dietary patterns (diets that are high in vegetables, fruit, whole grains, legumes and nuts) are beneficial. In turn, foods that promote weight gain (e.g., sugar-sweetened beverages), along with red and processed meats and alcohol, should be minimised—alcohol consumption is not only a contributor to caloric intake but also a recognised carcinogen.^17^

### Weight management

Focus on weight management enables a lifestyle pattern combining diet quality and quantity, alcohol intake and physical activity to be promoted and tested. Given the tendency for lifestyle behaviours to cluster/co-occur,^71^ implementation of equitable interventions that impact on several key areas of lifestyle offer considerable scope for reducing the overall disease burden. Although many unanswered questions exist within lifestyle interventions, with respect to dose, duration, type (for physical activity), caloric composition and diet quality (in terms of food intake), and how best to support long-term adherence, there is much that we can learn from longer-term lifestyle trials including those focusing on diabetes prevention. For example, intervention design no longer focuses on knowledge exchange alone, but integrates goal-based behavioural interventions, the use of lifestyle coaches, frequent contact and support and ‘toolbox strategies' to enable individual tailoring.^72^ Furthermore, recent work has highlighted the impact of using behavioural change techniques to support changes in diet and physical activity.^73^

## Weight-loss trials—challenges and opportunities

**Fig. 1:**
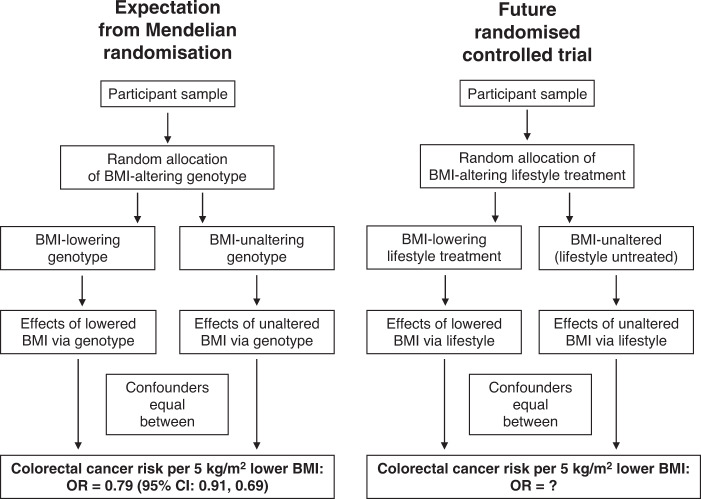
Expected effects of lowering BMI on cancer risk—how Mendelian Randomisation can guide research. Current estimates from genetically informed Mendelian randomisation (MR) studies can be used to set expectations for the results of future randomised controlled trials. A recent meta-analysed MR estimate of BMI for colorectal cancer (from Jarvis et al.^76^) suggests that a 5 kg/m^2^ lower BMI would reduce the risk of developing colorectal cancer by ~20%. This MR estimate reflects lifetime exposure to this relatively lower BMI, and so the magnitude of reduced colorectal cancer risk in response to short-term BMI reduction is expected to differ.

The potential for ‘megatrials’ to answer nutritional questions has been described by Trepanowski and Ioannidis^74^ to address challenges such as selective reporting, small sample size, short length of follow-up and high costs (trials of non-pharmacological interventions are generally publicly funded, with relatively low budgets, which makes large sample sizes and lengthy follow-up protocols prohibitive). These challenges are common in nutritional trials (as with other clinical areas), and it is clear that the methodological rigour of complex dietary behavioural trials needs to improve. In reality, large randomised controlled trials are likely to improve our understanding of the impact of weight management on cancer risk, but will need to be considered alongside other data sources such as pooled cohort studies,^75^ triangulated MR approaches (see Fig. 1)^76^ and network meta-analysis.^77^ The science of trial design^78^ now offers a much clearer pathway for designing and addressing trial challenges, enabling researchers to optimise recruitment from populations of interest, incorporate intervention features (content, implementation, fidelity and adherence), comparator groups, adaptive trial design^79^ and to collect long-term outcomes. The key here is to assess the body of evidence appropriately by recognising the inherent weaknesses in the various research designs that contribute to it.

Although three decades of trials of behavioural weight-loss programmes such as the Diabetes Prevention Programme have successfully demonstrated a significant reduction in the incidence of diabetes, weight -loss programmes for cancer prevention have not received much funding. A 21st- century rationale (as described by Ballard et al.^80^) for this lack of investment points to a lack of good interim biomarkers, the need for prohibitively large sample sizes, uncertainties about life stage and appropriate ‘dose’ of intervention, the need to achieve sustained behaviour change and the apparent desire for genetic discoveries. There are also concerns that people who attempt and fail to adhere to weight-loss regimens might experience negative emotional responses and, indeed, self-blame if a subsequent diagnosis of cancer is made. However, the past decade has seen a portfolio of weight-loss regimens combining novel dietary approaches, motivational technologies and implementation science approaches, which will help to optimise adherence and provide supportive behaviour change strategies for weight-loss trials.^81,82^ Although multicomponent interventions offer significant challenges, such approaches have been successfully tested in diabetes^83^ and cognitive function^84^ contexts, and are feasible to implement. Modern wearable technologies to motivate and support behaviour change, remote objective data collection and record linkage to routine clinical or registry data for follow-up (of at least a decade) make some of the difficulties in cancer-prevention trials more manageable. Furthermore, improvements in trial design, understanding of intervention content and dose and knowledge regarding the provision of effective long-term support for behaviour change make successful cancer-prevention trials increasingly plausible. Nevertheless, an important challenge for primary prevention trial design is the identification of clinically meaningful short- and longer-term health outcomes. The search for robust and clinically relevant surrogate markers (e.g., adenoma recurrence in colorectal cancer, mammographic density and hormone levels in breast cancer) continues, and such markers would add considerable confidence to expensive intervention studies with long-term follow-up. However, it is also important to note that studies of chemoprevention (e.g., aspirin) that have cancer development as their primary outcome have been funded, and lifestyle interventions could do likewise.

### Weight management and high-risk populations

One notable population of interest for weight-management trials includes people who are known to be at a higher risk of developing cancer, including those with a family history of colorectal or breast cancer who are already undergoing surveillance procedures. In a large international multicentre trial of aspirin in patients with Lynch syndrome (hereditary non-polyposis colorectal cancer), Movahedi et al.^85^ reported that participants with obesity were 2.41 times (95% CI, 1.22–4.85) more likely to develop colorectal cancer than participants with under- and normal weight, and their risk increased by 7% for each 1 kg/m^2^ increase in BMI. There is considerable interest in weight management in women with a family history of breast cancer, although the greatest efforts to date have focussed on physical activity interventions. Gramling et al.^86^ reported from the Women’s Health Initiative observational study that healthy lifestyles (i.e., regular exercise, healthy body weight on the basis of BMI and <7 alcoholic drinks per week) led to a reduction in the risk of breast cancer in postmenopausal women, and the degree of this benefit was similar for women with and without a family history of breast cancer. A review by Pettapiece-Phillips et al.^87^ reported evidence of a protective role of a healthy body size and regular physical activity among *BRCA* mutation carriers, notably in adolescence and early adulthood. A number of feasibility or pilot trials of weight management have been undertaken in this high-risk population, including an assessment of the Diabetes Prevention Programme (with modifications) on breast cancer risk biomarkers.^88^ Intervention studies involving diet and physical activity,^89^ intermittent energy restriction,^90^ endurance training and nutrition counselling on the Mediterranean diet^81^ in individuals at increased risk of breast cancer are currently underway. These developmental studies point to the feasibility of initially ‘testing’ complex intervention trials in high-risk populations and should provide both rational and relevant platforms for planning definitive average-risk population-level randomised controlled trials.

## Conclusions

The need for much greater investment in research into cancer prevention is beyond question, and yet the current expenditure is only around 3% of the UK cancer research budget.^91^ Worldwide, excess weight is associated with the development of at least 480,000 new cancer cases each year.^7^ The bulk of current observational evidence on weight loss and obesity-related cancers suggests that decreasing body weight, reducing EBF and maintaining losses, by even relatively modest amounts, can have an impact on future cancer risk. It is important to note that most obese people who lose weight will remain in the obese category, but will have reduced cancer risk by even modest weight loss per se, which should therefore increase motivation for participating in interventions. However, clear gaps remain in our understanding of how changes in body fat or increased levels of physical activity are mechanistically linked to a decreased incidence of cancer. In addition, understanding the impact of different measures of EBF (e.g., body mass index, central obesity as assessed by waist circumference, bioelectrical impedance and DXA) adds to the complexity of identifying possible solutions.^11,12,92^ These gaps need to be urgently addressed to expedite the development and implementation of future cancer-control strategies.

Well-designed trials, providing robust evidence of impact, are crucial for efforts to garner funding for weight-management programmes aimed at reducing cancer risk. To date, trials of weight management and cancer prevention have almost exclusively been confined to feasibility work. The time has come for an international commitment to decreasing cancer burden and this commitment includes the development of large-scale intervention trials of weight management for primary prevention of obesity-related cancer—a point also raised in the paper on critical research gaps and recommendations in colorectal cancer.^93^ This need is urgent and the time to act is now!

## Data Availability

Not applicable.
